# Structure and the Anticancer Activity of Vitamin D Receptor Agonists †

**DOI:** 10.3390/ijms25126624

**Published:** 2024-06-16

**Authors:** Agnieszka Powała, Teresa Żołek, Geoffrey Brown, Andrzej Kutner

**Affiliations:** 1 Department of Organic and Physical Chemistry, Faculty of Pharmacy, Medical University of Warsaw, 1 Stefana Banacha, 02-097 Warsaw, Poland; 2 School of Biomedical Sciences, Institute of Clinical Sciences, College of Medical and Dental Sciences, University of Birmingham, Edgbaston, Birmingham B15 2TT, UK; g.brown@bham.ac.uk; 3 Department of Drug Chemistry Pharmaceutical and Biomedical Analysis, Faculty of Pharmacy, Medical University of Warsaw, 1 Stefana Banacha, 02-097 Warsaw, Poland; andrzej.kutner@wum.edu.pl

**Keywords:** vitamin D, vitamin D receptor (VDR), VDR ligand-binding domain, vitamin D metabolites and analogs, vitamin D anticancer analogs, calcitriol

## Abstract

Vitamin D is a group of seco-steroidal fat-soluble compounds. The two basic forms, vitamin D_2_ (ergocalciferol) and vitamin D_3_ (cholecalciferol), do not have biological activity. They are converted in the body by a two-step enzymatic hydroxylation into biologically active forms, 1α,25-dihydroxyvitamin D_2_ [ercalcitriol, 1,25(OH)_2_D_2_] and 1α,25-dihydroxyvitamin D_3_ [calcitriol, 1,25(OH)_2_D_3_], which act as classical steroid hormones. 1,25(OH)_2_D_3_ exerts most of its physiological functions by binding to the nuclear vitamin D receptor (VDR), which is present in most body tissues to provide support to a broad range of physiological processes. Vitamin D-liganded VDR controls the expression of many genes. High levels of 1,25(OH)_2_D_3_ cause an increase in calcium in the blood, which can lead to harmful hypercalcemia. Several analogs of 1,25(OH)_2_D_3_ and 1,25(OH)_2_D_2_ have been designed and synthesized with the aim of developing compounds that have a specific therapeutic function, for example, with potent anticancer activity and a reduced toxic calcemic effect. Particular structural modifications to vitamin D analogs have led to increased anticancer activity and reduced calcemic action with the prospect of extending work to provide future innovative therapies.

## 1. Introduction

Vitamin D was discovered over a century ago by Ellmer McCollum from investigations into the nature of rickets. Cod liver oil had been used in folk medicine to prevent and treat rickets and the substance McCollum isolated from cod liver oil that was deficient in rickets patients and that could prevent rickets curvature was named vitamin D. Subsequently, it became clear that vitamin D plays a primary role in the regulation of calcium-phosphate homeostasis and bone metabolism [1,2].

Importantly, the main form of vitamin D (cholecalciferol) in the human body results from a non-enzymatic transformation of 5,7-dehydrocholesterol in human skin when exposed to UV-B radiation (290–320 nm) [3,4,5]. Vitamin D_2_ and vitamin D_3_ become active when hydroxylated in the body by a two-step enzymatic process that leads to the production of 1α,25-dihydroxyvitamin D_2_ [ercalcitriol, 1,25(OH)_2_D_2_] and 1α,25-dihydroxyvitamin D_3_ [1,25(OH)_2_D_3_, calcitriol]. 1,25(OH)_2_D_3_ is a steroid hormone, as opposed to being a vitamin, because its systemic action parallels that of the classic steroid hormones, for example, testosterone, oestradiol, and progesterone [6,7]. 1,25(OH)_2_D_3_ largely exerts its physiological roles by binding to its specific nuclear vitamin D receptor (VDR) within cells, which, in turn, regulates the expression of a considerable number of genes [8,9]. The VDR is present in many tissues of the body, including those not originally recognized as target tissues for 1,25(OH)_2_D_3_. The dysfunction of VDR has been associated with hyperproliferative diseases, such as psoriasis and cancer, and observed for rickets, renal osteodystrophy, and autoimmune diseases such as multiple sclerosis, rheumatoid arthritis, inflammatory bowel disease, and type I diabetes [10]. With the discovery of VDR within extra skeletal cells, knowledge of the wide-ranging effects of vitamin D on other body systems has increased. 1,25(OH)_2_D_3_ promotes cell differentiation and induces apoptosis of cancer cells. It is involved in protecting cells against oxidative stress and regulating blood pressure. Vitamin D significantly benefits the immune system, including promoting the body’s non-specific defense against microbial infection and inhibiting the autoimmune response [2,11].

High levels of 1,25(OH)_2_D_3_ in the body lead to an increase in blood calcium, which can lead to soft tissue calcification and increased bone resorption [12]. Therefore, attention has been focused on synthesizing 1,25(OH)_2_D_3_ and 1,25(OH)_2_D_2_ analogs that retain the beneficial properties of the active forms of vitamin D and have an insignificant hypercalcemic effect [13,14]. This review examines how vitamin D analogs have been modified to reduce their calcemic activity and importantly retain and even increase their anticancer activity.

## 2. Metabolism of Vitamin D

### 2.1. Metabolism

Cholecalciferol is produced in the outer layers of human skin from 7-dehydrocholesterol, a major sterol derivative of cholesterol. Initially, 7-dehydrocholesterol is converted to the thermodynamically unstable pre-vitamin D_3_, which spontaneously isomerizes to vitamin D_3_. Unusually, this is a non-enzymatic reaction that requires energy supplied by UV-B radiation [15]. If overexposed to UV-B radiation, pre-vitamin D_3_ can be converted to inert products, such as tachysterol and lumisterol, to avoid the excessive production of vitamin D_3_ [16,17]. UV-B radiation is also used to generate ergocalciferol from membrane ergosterol [18].

Both vitamin D_3_ and vitamin D_2_ are also ingested via the diet and through the use of dietary supplements. These forms of vitamin D have no biological activity and are activated in the body by two-step enzymatic hydroxylation [5,19]. They are first transported by the vitamin D binding protein (VDBP) from the skin and enterocytes to the liver. There, they undergo initial hydroxylation at the C-25 position of vitamin D via the action of the liver enzymes 25-hydroxylase CYP2R1 and CYP27A1, leading to the formation of the circulating forms 25-OH-D_2_ and 25-OH-D_3_, respectively. The serum concentrations of these are measured in tests of vitamin D levels in the body [5,20,21,22]. The second hydroxylation, at C-1, occurs via the CYP27B1 enzyme, a 1α-hydroxylase found mainly in the kidney, resulting in the formation of the most biologically active forms 1,25(OH)_2_D_2_ and 1,25(OH)_2_D_3_. CYP27B1 is also present in extra-renal tissues, including skin, parathyroid glands, and skeletal, cardiovascular, and immune system cells [19,23,24]. The kidney enzyme CYP24A1 is responsible for the catabolism of the most active form of vitamin D and its precursor 25-OH-D to 1,24,25(OH)_3_D_3_ as the inactivation product, which is ten times less active than the most active forms.

### 2.2. Homeostasis

1,25(OH)_2_D_3_ levels in the body are internally regulated by the stimulation or inhibition of the transcription of the genes that encode the CYP27B1 and CYP24A1 enzymes under the influence of calcium and phosphorus ions, fibroblast growth factor 23 (FGF23), parathyroid hormone (PTH), and 1,25(OH)_2_D_3_ itself. PTH is responsible for inducing the transcription of the gene encoding the CYP27B1 enzyme and inhibiting the expression of the gene encoding the CYP24A1 enzyme. 1,25(OH)_2_D_3_ is involved in regulating PTH production and secretion by inhibiting the expression of the gene encoding this protein [5,25]. Elevated blood levels of 1,25(OH)_2_D_3_ inhibit PTH secretion, resulting in the inhibition of CYP27B1 activity and a decrease in 1,25(OH)_2_D_3_ levels. Calcitriol stimulates the intestinal and renal absorption of calcium and phosphate, the excessive absorption of which leads to hypercalcemia and hyperphosphatemia. Increased concentrations of calcium and phosphate ions in the blood inhibit the activity of CYP27B1. Calcium ions inhibit PTH, unlike phosphate ions, which do not, resulting in decreased 1,25(OH)_2_D_3_ concentrations. A decrease in the concentration of these ions leads to increased PTH levels, the activation of CYP27B1, and an increase in the synthesis of 1,25(OH)_2_D_3_ [26].

Unlike PTH, 1,25(OH)_2_D_3_ and FGF23 inhibit the expression of the gene encoding CYP27B1 and induce the transcription of the gene encoding CYP24A1. 1,25(OH)_2_D_3_ also stimulates the production of FGF23. The final effect of FGF23 is the inhibition of the synthesis of 1,25(OH)_2_D_3_, resulting in a decrease in FGF23 levels. The expression of these enzymes in healthy target tissues other than the kidney is low. CYP27B1 does not respond to the action of FGF23, PTH, or 1,25(OH)_2_D_3_ as it does in the kidney. Regulation of the expression of the gene encoding CYP24A1 only occurs under the action of 1,25(OH)_2_D_3_ as a protective mechanism in rare cases of toxic levels of 1,25(OH)_2_D_3_ in the blood [5,7,27,28,29,30,31].

## 3. Structure of Active Forms of Vitamin D

**Figure 1:**
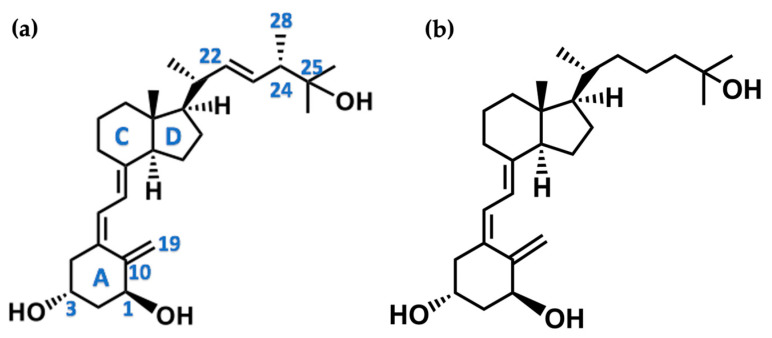
Chemical structures of the most active form of vitamin D_2_, 1,25-dihydroxyvitamin D_2_ (**a**), and vitamin D_3_, 1,25-dihydroxyvitamin D_3_ (**b**).

The active forms of vitamins D_2_ and D_3,_ 1,25(OH)_2_D_2_ and 1,25(OH)_2_D_3_ contain the same structural fragments: a cyclohexane A-ring with hydroxyls attached at C-1 and C-3 and methylene 19-CH_2_ at C-10, a conjugated triene system linking the A and CD rings, CD rings, and a side chain with hydroxyl at C-25. The A-ring can exist in two chair-like conformations, α and β. In these conformations, the hydroxyl at C-3 is oriented above or below the plane of the A-ring, respectively (Figure 1) [32]. The conformation of the A-ring has a significant effect on the interaction of the vitamin D compound with VDR. In binding with VDR, the A-ring adopts a β-chain conformation in which the hydroxyl group at C-1 adopts an equatorial orientation and the hydroxyl group at C-3 has an axial orientation. This arrangement allows the formation of strong hydrogen bonds between the hydroxyls of the A-ring and the amino acid residues of VDR. The A-ring also adopts a β-chair conformation when the compound is in the solid state and not bound to the VDR. This is due to the direct hydrogen bonds between the hydroxyl at C-1 and the hydroxyl at C-3 of the A-ring. (24*R*)-1,24(OH)_2_D_3_ (tacalcitol) is one of the few vitamin D analogs in which the α-chair conformation of the A-ring has been observed in the solid state. This is due to the indirect formation of hydrogen bonds between the hydroxyls of the A-ring involving water molecules [32]. The CD ring system is derived from a steroid precursor and its presence is not crucial for the vitamin D compound to show biological activity [33,34,35].

The most important element in the structure of the five-carbon aliphatic side chain is the hydroxyl at C-25, which plays an important role in the binding of the most active form of vitamin D to the VDR. 1,25(OH)_2_D_2_ and 1,25(OH)_2_D_3_ have minor differences in the side chain structure (Figure 1). The side chain of 1,25(OH)_2_D_2_ contains an additional double bond at C-22 and 28-CH_3_ at C-24.

## 4. Mode of Action of Active Vitamin D

### 4.1. Binding Site in the Cell—The Vitamin D Receptor (VDR)

1,25(OH)_2_D_3_ exerts most of its physiological functions by binding to its specific nuclear receptor, the VDR, which is involved in regulating the expression of several genes that encode mainly proteins responsible for calcium-phosphate and bone tissue metabolism. These include osteopontin, osteocalcin, collagen, the parathyroid hormone (PTH), Na^+^/phosphate cotransporter NaPi-2b/Slc34a2, calcium transporters (renal TRPV5 and intestinal TRPV5 and TRPV6), the RANK receptor and its ligand RANKL, alkaline phosphatase, calbindin 1 (CALB1), 1α-hydroxylase, and CYP24A1 [9,36,37]. VDR is also responsible for regulating the expression of genes encoding proteins involved in completely different physiological processes, such as the growth hormone, the insulin receptor, complement components, cytochrome P450, CYP3A4, cAMP, CD14, FBP1, and renin [7,38]. 1,25(OH)_2_D_3_ target genes can be divided into two groups. The first is the so-called primary target genes, whose expression is directly regulated by the 1,25(OH)_2_D_3_-liganded VDR. The second group consists of so-called secondary target genes, whose expression is regulated by transcription factors encoded by the main vitamin D target genes. This group of transcription factors includes BCL6, NFE2, POU4F2, ELF4, IRF5, MAFF, MYCL, NFXL1, SRA1, and others [5,9,38,39].

In vitro studies have shown that 1,25(OH)_2_D_3_ mostly inhibits the expression of its target genes, especially when cells are stimulated with 1,25(OH)2D_3_ for 24 h or more. Inhibition of the expression of a particular gene by 1,25(OH)_2_D_3_ is only possible if gene expression is stimulated by other transcription factors. Blocking the action of one or more transcription factors that induce the expression of a particular gene is the most likely explanation for the mechanism of inhibition of target gene transcription by 1,25(OH)_2_D_3_. Consequently, most target genes regulated this way should be classified as indirect targets since 1,25(OH)_2_D_3_ counteracts rather than directly inhibits their expression. To summarize the described mechanism of action of vitamin D, 1,25(OH)_2_D_3_, it directly induces or inhibits the expression of VDR-mediated target genes or prevents their induced expression via other transcription factors [38,40].

### 4.2. Non-Genomic Action

1,25(OH)_2_D_3_ exerts its action mainly by interacting with the VDR to regulate the transcription of genes [9]. The idea of alternative pathways that are activated by 1,25(OH)_2_D_3_ arose from a rapid (1–10 min) calcium influx induced by 1,25(OH)_2_D_3_ as seen from studies using ROS 17/2.8 osteogenic sarcoma cell lines and isolated myocytes from embryonic chicken hearts. Subsequently, the activation of rapid extra genomic responses to 1,25(OH)_2_D_3_ was observed in in vivo chicken studies, cultured mouse chondrocytes lacking VDR, and the osteoblastic ROS 24/1 cell line that lacks VDR expression.

Thus, the existence of an extranuclear receptor was postulated for 1,25(OH)_2_D_3_, which was responsible for its effect on the instantaneous change in calcium content in cellular compartments, as well as on membrane channel function, prostaglandin metabolism, the catabolism of cell membrane lipids, regulation of the amount of phosphoinositol and cGMP in the cytoplasm, the activation of enzymes present in the cytoplasm (protein kinase C, Ras kinase, mitogen-activated kinase-MAPK, and sphingomyelinase), and the pathway mediated by Wnt proteins. These are agents that control many physiological and pathological processes, such as embryogenesis and beta-catenin, which influence the maintenance of the normal structure of intercellular junctions, regulate the cytoskeleton, and can also act as a transcription factor.

A proposed extranuclear receptor for 1,25(OH)_2_D_3_ is the membrane-bound immediate-response steroid-binding protein 1,25-D3-MARRS, also known as PDIA3, GRP58, or ERp57. The binding of 1,25(OH)_2_D_3_ to the PDIA3 protein leads to an immediate increase in calcium and phosphorus absorption in intestinal cells [41,42]. From in vitro studies using squamous cell carcinoma cell lines, the deletion of PDIA3 altered the expression of genes that are involved in the regulation of bone mineralization, phospholipase C activity, and calcium-dependent phospholipid binding. In addition, in vivo studies in mice showed that partial silencing of PDIA3 (PDIA3+/−) impaired skeletal development in experimentally treated animals, whereas PDIA3 deletion was lethal to these animals [43,44,45,46].

Another protein identified as an extranuclear receptor for 1,25(OH)_2_D_3_ is annexin II, which affects endocytosis, exocytosis, and cell membrane structure [39]. The nuclear VDR may also be involved in the extra genomic action of 1,25(OH)_2_D_3_, as evidenced by its presence in the caveolae-rich areas of the cytoplasm [36,39,47]. Recent studies on A431 squamous cell carcinoma cells have shown that both the VDR and PDIA3 are required in the regulation of the membrane response to active forms of vitamin D [46].

## 5. Biological Activity of Vitamin D and Its Metabolites

1,25(OH)_2_D_3_ primarily affects the development and metabolism of bone tissue and the maintenance of normal blood calcium and phosphate levels by inducing their absorption in the gastrointestinal tract and kidneys. 1,25(OH)_2_D_3_ is responsible for the proper functioning of the skeletal system by influencing the maintenance of adequate calcium and phosphorus levels in the blood. In addition, the VDR associated with the active metabolite of vitamin D induces the transcription of genes encoding bone proteins (osteocalcin and osteopontin), calcium-binding proteins CaBP and IGFBP-3, and the proteins involved in intracellular calcium transport and phospholipid metabolism. 1,25(OH)_2_D_3_ also acts on bone cells, influencing the balance between bone resorption and bone formation. Its anti-apoptotic effect on osteoblasts, which are responsible for producing the organic part of the bone matrix, influences the increase in bone mass. 1,25(OH)_2_D_3_ deficiency in the body causes a decrease in the mechanical strength of the skeletal system [48]. An important action of 1,25(OH)_2_D_3_ is its effect on reducing parathyroid cell proliferation and PTH secretion to inhibit bone resorption [7].

1,25(OH)_2_D_3_ has a significant immunomodulatory effect on the innate and acquired elements of the immune system, as evidenced by the presence of VDR in a large number of immunologically active cells, mainly macrophages, dendritic cells, and activated T lymphocytes. By binding to VDR, 1,25(OH)_2_D_3_ induces the expression of genes encoding the so-called natural antibiotics, i.e., cathelicidin and defensin proteins [49]. The results of epidemiological studies confirmed an association between living in areas with limited access to sunlight for people living in northern geographical areas and a higher incidence of autoimmune diseases such as rheumatoid arthritis, type I diabetes, inflammatory bowel disease, or multiple sclerosis [50]. Such observations have been linked to vitamin D’s ability to immunosuppress, including by reducing the levels of T-helper cytokines in the body. Multiple sclerosis is an important autoimmune disease, and the risk of occurrence has been linked to vitamin D deficiency. 1,25(OH)_2_D_3_ inhibits the activity of CD4+ T lymphocytes and autoreactive MPB-specific lymphocytes, resulting in a reduction in immune system activity. In addition, the active metabolite of vitamin D positively affects the development of cells responsible for IL-10 production, while reducing the number of cells secreting IL-6, IL-7, and IFN-γ [51]. T lymphocytes can also express CYP27B1 [52]. In autoimmune dermatological diseases, the effects of vitamin D on the immune system are of great importance. 1,25(OH)_2_D_3_ induces the expression of the cytokine TSLP, which plays a key role in atopic dermatitis and increases the number of Treg lymphocytes, which have immunosuppressive effects that can inhibit cutaneous allergic reactions [7]. Vitamin D deficiency has also been observed in patients with established rheumatoid arthritis (RA) [53]. 1,25(OH)_2_D_3_ reduces the number of Th1 and Th17 lymphocytes, which are considered pathogenic in RA, increases the number of Th2 and Treg cells, which quench the immune response, and reduces the production of the pro-inflammatory cytokines IL-1, -2, -6, -12, -17, or TNF-α while increasing the secretion of the anti-inflammatory cytokines IL-4, -5, and -10. The presence of the VDR and 1,25(OH)_2_D_3_ has been observed in chondrocytes, synoviocytes, and macrophages found in the joints of RA patients. 1,25(OH)_2_D_3_ plays an important role in preventing infection. This is related to VDR expression by antigen-presenting macrophages, dendritic cells, and CD4 and CD8 T lymphocytes [7]. 1,25(OH)_2_D_3_ supports the action of monocytes and macrophages against tuberculosis by increasing the level of the cathelicidin antimicrobial peptide or the membrane-anchored glycoprotein cluster of differentiation 14 (CD14), which functions as a co-receptor for Toll-like receptors [17].

1,25(OH)_2_D_3_ affects the cardiovascular system via the presence of the VDR and 1α-hydroxylase in cutaneous capillaries, cardiac myocytes, and fibroblasts, as well as in endothelial and vascular smooth muscle cells. They are involved in the expression of genes encoding structural proteins and vascular endothelial growth factors, matrix metalloproteinase 9, myosin, and several proteins that control normal blood pressure [7,54,55,56]. 1,25(OH)_2_D_3_ also directly affects the renin–angiotensin–aldosterone system by reducing renin and angiotensin levels in the blood coagulation system. For human leukemia cells and monocytes, 1,25(OH)_2_D_3_ increased the amount of the thrombomodulin protein to inhibit coagulation and inflammatory processes. Several studies involving large groups of subjects have shown that people with low blood levels of 1,25(OH)_2_D_3_, caused in part by a lack of vitamin D in their diet, have an increased risk of cerebral circulatory disorders that can lead to disease, including stroke. The results obtained from a study of 130 patients with known hypertension who were given cholecalciferol at a dose of 3000 IU/day for 20 weeks showed the effect of the 1,25(OH)_2_D_3_ in lowering their systolic blood pressure. However, there is still a lack of randomized clinical trials to confirm the role of vitamin D in preventing cardiovascular disease. In vivo experiments in laboratory animals provided important data on this issue. In experiments using mice lacking the VDR gene, increased renin expression, hypertension, and myocardial hypertrophy were observed in the animals [7].

1,25(OH)_2_D_3_ affects cancer cells. 1,25(OH)_2_D_3_ binds to the VDR to regulate the transcription of genes encoding proteins responsible for cell differentiation, proliferation, and apoptosis. 1,25(OH)_2_D_3_ inhibited the proliferation of tumor cells. For SCC-25 head and neck squamous cell carcinoma cells, the inhibition of cell cycle progression was seen from the accumulation of cells in the G0/G1 phases of the cell cycle. There was also increased expression of the DNA repair factor GADD45a at the mRNA and protein levels after treatment of cells with 1,25(OH)_2_D_3_. Similar effects have been observed in studies on the effect of calcitriol on cultured human melanoma cells [57]. For human breast cancer MCF-7 cells, 1,25(OH)_2_D_3_ repressed expression of the c-myc protooncogene and increased expression of the c-myc antagonist, the MAD1/MXD1 protein. Treatment of the human colon and rectal cancer cells SW620, PC/JW, and HT-29 with 1,25(OH)_2_D_3_ and its analog, seocalcitol, led to a p53-independent induction of apoptosis, G1-phase cell cycle inhibition, and an increase in the pro-apoptotic protein Bak. 1,25(OH)_2_D_3_ had a negative effect on human LNCaP prostate cancer cells and SW-480 colon cancer cells. 1,25(OH)_2_D_3_-mediated inhibition was observed regarding the secretion of VEGF, endothelin 1, and glucose transporter 1, which act as fundamental elements in angiogenesis [7,58].

Animals lacking the VDR develop larger-diameter vessels that then supply blood to tumors. Studies using triple-negative breast cancer cells have demonstrated the role of the nuclear protein cathepsin L as a biomarker, whereby an elevated level of expression correlated inversely with the repair protein 53BP1 when VDR receptor levels were low. 1,25(OH)_2_D_3_ showed antiproliferative and differentiation effects on human promyelocytic leukemia cell lines (HL-60, MV4–11) [59]. Ongoing studies are aimed at further developing our knowledge of the potential of 1,25(OH)_2_D_3_ and its synthetic analogs in the very important field of cancer therapy [7].

## 6. Vitamin D Receptor 

### 6.1. Structure of the Nuclear Receptor Family

**Figure 2:**
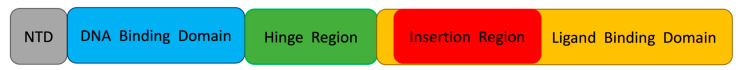
The domain structure of the vitamin D receptor consists of the N-terminal domain (NTD), the DNA-binding domain, the hinge region, the insertion region, and the ligand-binding domain.

The VDR receptor belongs to a nuclear receptor (NRs) family. These receptors act as ligand-dependent transcription factors regulating gene expression, which is responsible for cell growth and differentiation, the maintenance of homeostasis, and a variety of other physiological processes [8,9,38,60]. The VDR is the only member of the NR1H/NR1I subfamily that evolved into an endocrine receptor that accepts its specific ligand, 1,25(OH)_2_D_3_, at subnanomolar concentrations [9]. NRs share a common structural organization comprising a flexible, variable N-terminal domain containing the ligand-independent AF-1 activation domain, a DNA-binding domain (DBD), and a C-terminal ligand-binding domain (LBD) containing the ligand-dependent AF-2 activation domain. The DBD and LBD domains are connected by a hinge region (Figure 2) [8,61].

Unlike other NRs, the VDR does not contain an AF-1 activation domain in its structure. VDR LBD contains a long fragment located in the region connecting helix H1 to helix H3. This region has a disordered structure. To the best of our current understanding, it does not play a significant role in ligand–receptor binding [62]. Another characteristic feature of NRs is their ability to interact with numerous groups of compounds, such as DNA sequences or protein cofactors, resulting in various biological effects. Most NRs function as homo- or heterodimers with other NRs. VDR forms a heterodimer with one of the three isotopes of the retinoid X receptor (RXRα, NR2B1, RXRβ, NR2B2, or RXRγ, NR2B3) and in this form, binds to specific DNA sequences within the promotors of target genes (VDRE). The VDR also interacts with VDREs as a homodimer. The crystal structures of VDR-LBD and VDR-DBD were obtained [8,61,63,64,65].

### 6.2. Structure of the Ligand-Binding Domain (LBD)

The ligand-binding domain (LBD) is a dynamic structure that undergoes stabilization upon the binding of ligands [66,67]. The LBD consists of a triple-layered α-helix sandwich consisting of 12 helices (H1-H12), three double helices (H3n, H4n, and Hx), and a triple-layered β-helix [8]. Ligands bind to the receptor at a site called the receptor binding pocket (LBP). The LBP is surrounded by the helices H2, H3, H5, H6, H7, H10, and H12 and is predominantly composed of hydrophobic amino acid residues. The amino acid residues of each β-harmonic strand also form links with the ligand. The amino acid residue Trp-286 is a VDR-specific amino acid in the β1 band that interacts with the ligand.

**Figure 3:**
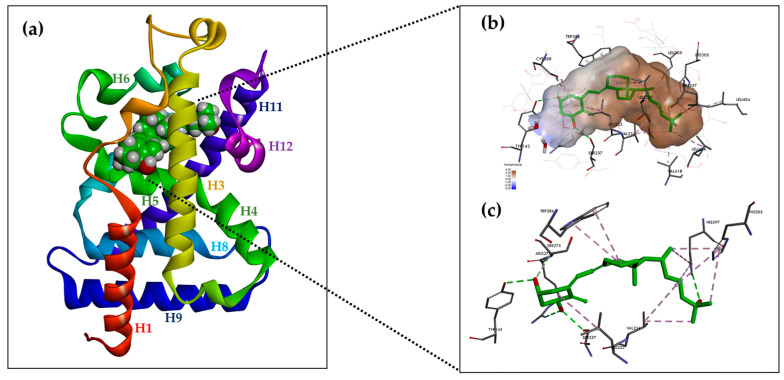
Structure of hVDR LBD (RCSB PDB ID: 1DB1, adapted from Rochel N. et al. [63], created with DISCOVERY STUDIO v. 22 software (https://discover.3ds.com, accessed on 24 May 2024). (**a**) Ribbon representation of the VDR LBD bound to 1α,25(OH)_2_D_3_ (green color), composed of 12 helices. (**b**) Binding mode of 1,25(OH)_2_D_3_ in the VDR LBP. The volume of the LBP is shown as a hydrophobic surface. The interacting residues and their locations in the VDR structure are shown. (**c**) The residues anchored through specific H-bonds and hydrophobic interactions to 1,25(OH)_2_D_3_ are depicted.

The LBD also contains regions necessary for VDR heterodimerization with RXR (H9 and H10, as well as loops 8–9) and the AF-2 region. The AF-2 region allows VDR interaction with co-activators, as guided by helices H3, H4, and H12. Helix H12, which encapsulates the LBP, is stabilized by direct interactions via van der Waals interactions between the amino acid residues Val-418 and Phe-422 and the methyl of the ligand, a series of hydrophobic interactions between the residues Thr-415, Leu-417, Val-418, Leu-419, Val-421, and Phe-422 and the amino acid residues of helixes H3 (Asp-232, Val-234, Ser-235, Ile-238, and Gln-239), H5 (Ala-267 and Ile-268), and H11 (His-397 and Tyr-401), and two polar interactions between the Lys-264 (H4)-Glu-420 bridge and the hydrogen bond between Ser-235 (H3) and Thr-415. Some of the amino acid residues stabilizing the H12 position, Val-234 (H3), Ile-268 (H5), His-397 (H11), and Tyr-401 (H11), are in contact with the ligand, resulting in an additional indirect mechanism for the ligand to control the H12 position. The backbone carbonyls of Met-412 and Leu-414 (loop H11-H12) are linked by a hydrogen bond to the H2 end. Arg-154 forms a hydrogen bond with Asp-232 (H3) (Figure 3a) [8].

One characteristic of the LBD domain is the presence, in the protein connecting helices H1 and H3, of a region varying in length between 72 and 81 amino acid residues, which does not significantly affect ligand binding to the receptor, is subject to protease action, and contains a phosphorylation site that has no known function. The presence of this unregulated region has been an obstacle in attempts to crystallize the VDR LBD. The location of this region is distant from the amino acid residues involved in ligand binding to the receptor and the formation of the VDR-RXR heterodimer and, therefore, most likely, does not affect these interactions [8,62,63].

### 6.3. Arrangement of 1,25(OH)_2_D_3_ in the LBD VDR

The crystal structure of 1,25(OH)_2_D_3_-hVDR-LBD has been reported. A truncated form of hVDR was used to crystallize the hVDR-LBD-1,25(OH)_2_D_3_ complex. This lacks the disordered region in the connecting helices H1 and H3 [63]. Analysis of the crystal structure of the complex allowed the authors to investigate the behavior of 1,25(OH)_2_D_3_ interacting with the VDR. Surprisingly, 1,25(OH)_2_D_3_ occupies only 56% of the LBP volume, leaving room for modification and extension of the sidechain.

The A-ring of 1,25(OH)_2_D_3_ bound to the VDR adopts a β-chair conformation in which the C-19 methylene group is in the upper position and the hydroxyls at C-1 and C-3 of the A-ring adopt an equatorial and axial orientation, respectively. The α-chair conformation of the A-ring negatively affects the hydrogen bonds between the hydroxyls of the A-ring and the amino acid residues of the receptor and prevents the compound from nesting in the LBP due to the occurrence of steric clashes with Phe-150. Near the C-2 of the A-ring, the pocket space is occupied by two water molecules. The side chain at C-17 of the D-ring adopts an extended conformation parallel to the bond between C13 and C18 and is surrounded by hydrophobic amino acid residues. The conjugated triene system connecting the A and C rings is in a hydrophobic channel between Ser-275 (loop H5-beta) and Trp-286 (beta1) on one side and Leu-233 (H3) on the other. The non-planar geometry of the conjugated triene in the complex results in a curved ligand shape. The C-ring is in contact with Trp-286, and the C-18 methyl faces Val-234 (H3). The bond between C6 and C7 adopts a *trans* conformation that deviates 30 degrees from a planar geometry (Figure 3b).

The compound is held in the LBP by three pairs of hydrogen bonds formed between a hydroxyl located at C-1 in the A-ring and the polar amino acid residues of the receptor Ser-237 (H3) and Arg-274 (H5, the strongest interaction); between the hydroxyl located at C-3 in the A ring and the polar amino acid residues of the receptor -Ser-278 (H5) and Tyr-143 (loop H1-H2); and between the hydroxyl located at C-25 in the side chain and the polar amino acid residues of the receptor His-305 (loop H6-H7) and His-397 (H11) (Figure 3c). The hydrogen interactions are the most important anchoring elements of the ligand within the LBP and must, therefore, be maintained for ligand activity. The amino acid residue of Arg-274 forms a hydrogen bond with water molecules, resulting in a water channel. Much of the LBP remains free after binding to the ligand leaving a wide space, particularly around the side chain and C-2 at A-ring [8,63].

Numerous crystallographic X-ray diffraction structures of 1,25(OH)_2_D_3_ and synthetic analogs have been reported, including maxacalcitol, seocalcitol, calcipotriol, KH1060, MC1288, TEI-9647, *des*-CD analogs, carborane analogs, litocholic acid derivatives, Gemini analogs, and 25-nitro-1,25(OH)_2_D_3_ complexed with hVDR LBD. Based on the analysis of these structures, it was possible to observe the differences in binding to the VDR between 1,25(OH)_2_D_3_ and analogs.

### 6.4. DNA Binding Domain (DBD)

The DBD is the most conserved element in the structure, not only of the VDR but also of the other NRs. It consists of two zinc fingers, the first of which is involved in the binding of VDR-RXR to the specific DNA sequences of the genes controlled by the VDR, while the second affects the interaction of the heterodimer. The spherical constraints of the VDR-RXR complex define the optimal binding site of the heterodimer to the VDRE as a direct repeat of the RGKTSA sequence (R=A or G; K=G or T; S=C or G), separated by three nucleotides (DR3). Individual NR heterodimers differ in the number of nucleotides between the repeated sequences [8,68,69,70]. ChIP-seq studies on a variety of cell types have shown that DR3 accounts for only 10–20% of all VDR binding sites, with the majority of VDREs dispersed throughout the genome [8,38,71,72,73,74,75,76]. The low percentage of VDR complexes that bind to DR3 detected by ChIP-seq suggests that the VDR may act independently of RXRs by using other nuclear proteins as alternative cooperative binding partners to genomic DNA [8,38]. Additionally, VDR response regions (VDREs) can be located at a considerable distance from the coding region of the controlled genes [8,38,77].

### 6.5. Mode of Action of VDR

Various cell-specific transport proteins and microtubular elements of the cytoskeleton mediate ligand transport to the nucleus. Agonist binding to the VDR induces a conformational change in the VDR LBD in the AF-2 region, resulting in corepressor detachment and coactivator recruitment to the VDR-RXR heterodimer [78]. The recruitment of coactivators with histone acetyltransferase activity (HAT) prepares the promoters of target genes through chromatin decondensation. The HATs can then be replaced by a mediator complex that allows the heterodimer to bind to RNA polymerase II located at the transcription start site of a specific gene. Typically, in the absence of a ligand or the presence of an antagonist, the VDR remains bound to proteins with histone deacetylase activity that act as corepressors, preventing RNA polymerase II from accessing the relevant DNA sequences and resulting in the inhibition of transcription of the respective genes [77]. The commodulator proteins exhibit coactivator or corepressor properties depending on the cell type. The VDR can also form VDR2 homodimers and heterodimers with other nuclear receptors such as the retinoic acid receptor (RAR). There is cooperation between all-trans retinoic acid ATRA and vitamin D derivatives and the VDR-RAR heterodimer in the induction of the transcription of vitamin D-controlled genes, e.g., the gene encoding osteocalcin. Even so, the formation of the VDR-RXR heterodimer is the most important response of the VDR to a ligand regarding biological activity [8,39].

### 6.6. The Mechanism of Action of VDR Coregulators

Once the ligand binds to the VDR, the receptor interacts with several nuclear proteins to form a multi-protein complex. The complex includes coreceptors (RXR), pioneer factors (PU.1, CEBPα, GABPα, ETS1, RUNX2, and BACH2), chromatin-modifying enzymes (HATs, HDACs, KDM1A, and KDM6B), chromatin-remodeling proteins (BRD7 and BRD9), coactivators (MED1 and the NCOA family), and corepressors (NCOR1 and COPS2) [5,38,79,80,81,82].

The mechanism of action of coregulators and coactivators with the VDR is well understood, but the function of corepressors requires further research. Among the most important coactivators that bind to the VDR are NCOAs and mediator complexes. NCOAs (NCOA1, NCOA2, and NCOA3) mobilize the secondary coactivators CBP/p300 and p/CAF, which have histone acetyltransferase activity, and their binding to the receptor is dependent on the presence of 1,25(OH)_2_D_3_. MED1 is a VDR coactivator that acts as a linker between the protein complex of the activated VDR receptor and RNA polymerase II located at the transcription start site (TSS) of a specific gene [8,83,84,85].

The structures of most coactivators that interact with ligand-bound NRs contain the LXXLL motif, which forms an amphipathic α-helical structure that binds to the AF-2 region of the receptor, or the NR box, which is often found in the disordered regions of coactivators, but also of corepressors. Corepressors contain the LXXH/IIXXXI/L motif in their structure [86,87,88,89]. Nuclear coreceptors interact with NCOAs through a receptor interaction domain (RID) formed by three LXXLL motifs. Each domain in the NCOA structure is specialized for binding specific transcription factors or other co-regulators. The VDR preferentially interacts with the second and third LXXLL motifs of the NCOA receptor interaction domain. MED1 contains two NR boxes located in the central receptor interaction domain. The VDR interacts with the second motif [90,91]. Numerous crystal structures of LBD NRs in combination with LXXLL motif coregulators, including the VDR LBD, are available from the PDB database [8,92,93,94].

### 6.7. VDR-RXR Heterodimer—Allosteric Domain Interaction

Only the X-ray crystal structure of the VDR-LBD monomer has been solved. The full-length crystal structure of the VDR-RXR-LBDs heterodimer is still unknown, which may be due to the weaker affinity of the VDR for the RXR compared to the other NRs. Methods such as SAXS, cryo-EM, and HDX-MS play an essential role in providing the structure of the VDR-RXR complex in combination with, e.g., DNA, allowing the simultaneous visualization of how the DBD and LBD domains of the heterodimer are arranged in relation to each other and how their binding to the ligand, DNA, and coactivators affects each other [8,95,96]. LBDs and DBDs in the VDR-RXR complex are arranged asymmetrically, at some distance from each other. The linking regions of the domains play an important role in maintaining the integrity of the structure. The structure of the domain-linking region in RXR is not well defined, unlike the linking region in VDR, which is known to form an α-helix responsible for the integrity of the entire VDR-RXR complex, making it easier for coregulators to bind to the heterodimer [8,95,96].

An allosteric interaction between VDR LBD, VDR DBD, and DNA was shown by HDX-MS for 1,25(OH)_2_D_3_ interacting with the VDR-RXR LBDs complex. The VDR DBD and VDR LBD work together to coordinate the mechanisms of transcriptional regulation of ligands and DNA [97]. Ligand binding to the receptor leads to conformational changes in the VDR DBD and stabilizes the VDR-RXR heterodimer complex. The HDX profile of 1,25(OH)_2_D_3_ bound to the VDR-RXR complex in the presence of the RXR receptor ligand showed significant similarity to that of 1,25(OH)_2_D_3_ bound to the complex independently. Allosteric communication is ligand-dependent and bidirectional. The binding of the VDR-RXR heterodimer to the relevant DNA sequences triggers DBD-DBD and LBD-LBD interactions and affects coactivator binding. The binding of the VDR-RXR complex to the appropriate DNA fragment results in conformational changes in LBD in the coactivator interacting regions (VDR-H12 and RXR-H3) and the H10-H11 dimer binding site [8,97].

## 7. Synthetic Analogs of Vitamin D

The therapeutic potential of vitamin D compounds is of substantial interest worldwide. 1,25(OH)_2_D_3_ is a hormone that exerts a wide range of physiological effects, many of which are not yet fully understood. However, the antiproliferative and differentiating effects of 1,25(OH)_2_D_3_ on cancer cells are important to new and safer cancer treatments. Unlike conventional chemotherapeutic drugs, the only toxic, clinically relevant limiting factor of 1,25(OH)_2_D_3_ is the increased blood calcium level. This increase limits the use of effective therapeutic doses. Many analogs of 1,25(OH)_2_D_2_ and 1,25(OH)_2_D_3_ have been synthesized to elucidate a correlation between their structure and biological activity [13]. From various structural modifications, several analogs have retained the properties of 1,25(OH)_2_D_3_ and, importantly, have a lowered calcemic effect compared to 1,25(OH)_2_D_3_. There is still a continuous search for an analog that shows a specific physiological effect without any toxic effect. For example, there is still the need to synthesize analogs that have greater antiproliferative and cell-differentiating activity and exhibit much lower calcemic properties compared to 1,25(OH)_2_D_3_. They also need to be much more resistant to CYP24A1 catabolism than 1,25(OH)_2_D_3_.

### 7.1. Therapeutics in the Vitamin D Group

Several vitamin D metabolites and analogs are already used as therapeutics. Apart from vitamin D_2_ and D_3_, widely used as a therapeutic and dietary supplement, calcipotriol, as used against psoriasis, is the most important synthetic analog of 1,25(OH)_2_D_3_. Table 1 shows the most clinically important vitamin D compounds and the conditions that they are used to treat [13,14,98,99].

### 7.2. Anticancer Analogs of 1,25(OH)_2_D_2_ with Modified A-Ring and Side Chain

A considerable number of synthetic analogs of 1,25(OH)_2_D_3_ have been reported [13,14,99]. Table 2 lists the analogs of 1,25(OH)_2_D_2_ with particularly favorable anticancer activity. The affinity for the VDR and functional activity of the analogs were determined. In vitro studies using various human cancer cell lines have demonstrated the differentiation and antiproliferative activity of the analogs, and in vivo toxicity studies confirmed their decreased effect on increasing blood calcium concentration compared to 1,25(OH)_2_D_3_. The analogs belong to two groups. The first group consists of analogs with a single-site modified structure, PRI-1907, PRI-1906, and their (24Z) isomers, PRI-1917 and PRI-1916. The modified site of the analogs is their side chain rigidified by introducing the conjugated diene moiety delta-22,24 and extended by one carbon unit (24a-homo). In PRI-1907 and PRI-1917, the side chain is homologated at both terminal carbons. These analogs showed differentiation and antiproliferative activity against the human promyelocytic leukemia cell lines HL-60 and MV4–11. PRI-1917 showed lower biological activity compared to PRI-1907, whereas PRI-1916 showed greater biological activity compared to PRI-1906. PRI-1907 and PRI-1917 demonstrated antiproliferative activity against the human colon cancer cell line HT-29. In vitro studies using AML cell lines and syngeneic mouse models of AML in vivo have demonstrated the ability of PRI-1917 and PRI-1916 to cooperate at low concentrations with the plant polyphenol carnosic acid, and significantly enhanced the differentiation effect on cells. They inhibited the G1-to-S cell-cycle transition in a cell type-dependent manner. PRI-1906 enhanced the anticancer effect of cyclophosphamide in a mouse model of mammary carcinoma 16/C. PRI-1907 is the most active analog with a single-site modified structure. Interestingly, all analogs showed a lower affinity for the VDR compared to 1,25(OH)_2_D_3_. (24Z) isomers PRI-1917 and PRI-1916 had a lower affinity for the VDR compared to (24E) isomers PRI-1906 and PRI-1907, respectively.

The second group of analogs includes PRI-5201, PRI-5202, PRI-5105, and PRI-5106 with a double-site modified structure. PRI-5201 and PRI-5202 were modified via stiffening with a conjugated diene and the extension of their side chain by one carbon unit (24a-homo) and depletion of C-19 methylene at the A-ring. PRI-5202 was additionally homologated at both terminal carbons. The removal of 19-methylene led to a decrease in calcium action. PRI-5201 and PRI-5202 showed significant differentiation and antiproliferative activity against the human promyelocytic leukemia cell lines HL-60 and MV4–11, the breast cancer cell lines MCF-7 and T47D, and the colon cancer cell line HT-29. In addition, for the human ovarian cancer cell line (HGSOC), PRI-5202 was the most potent analog out of all tested lines (PRI-1906; PRI-1907; PRI-5201; PRI-5202), with the induction of CYP24A1 expression and reduction in cell number. The structural modification of PRI-5105 and its (24R) isomer PRI-5106 consisted of one-carbon homologation (20a-homo) and the introduction of a C22-C23 double bond in their side chain and removal of the C-19 methylene. PRI-5105 and PRI-5106 showed differentiation and antiproliferative activity against the human promyelocytic leukemia cell lines HL-60 and MV4–11. PRI-5105 and PRI-5106 showed greater antiproliferative activity compared to 1,25(OH)_2_D_3_ and significantly enhanced the antiproliferative effect of 5-fluorouracil against the human colorectal cancer cell line HT-29. However, both analogs showed undesirably low resistance to metabolic conversion by CYP24A1 compared to other analogs (PRI-1906; PRI-1907; PRI-5201; and PRI-5202) indicating that side chain stiffening, which is lacking in these analogs, is necessary for resistance. PRI-5201 and PRI-5202 showed greater affinity for the VDR compared to 1,25(OH)_2_D_3_. The crystallographic structures of these analogs complexed with hVDR LBD have not been determined yet. Docking experiments of these analogs to the VDR LBD are needed to elucidate how they interact with the VDR [59,100,101,102,103,104].

**Table 2:** Analogs of 1,25(OH)_2_D_2_ with anticancer activity.

Compound	Structure	Classification/Type of Cancer (Biological Activity)
PRI-1907	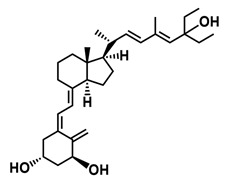	PC-3—prostate cancer cells (antiproliferative activity [105] HL-60, MV4–11—human promyelocytic leukemia cells (antiproliferative and differentiation activity) [59,100] HT-29—human colon cancer cells (antiproliferative activity) [59]
PRI-1917	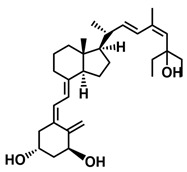	HL-60, MV4–11—human promyelocytic leukemia cells (antiproliferative and differentiation activity) HT-29—human colon cancer cells (antiproliferative activity)
PRI-1906	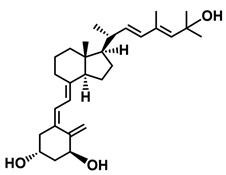	PC-3—prostate cancer cells (antiproliferative activity) [105] HL-60, MV4–11—human promyelocytic leukemia cells (antiproliferative and differentiation activity) [59] mouse mammary 16/C cancer model (antitumor activity in the combined therapy with CY) [104]
PRI-1916	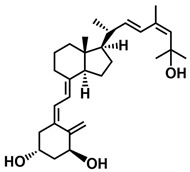	HL-60, MV4–11—human promyelocytic leukemia cells (antiproliferative and differentiation activity)
PRI-5201	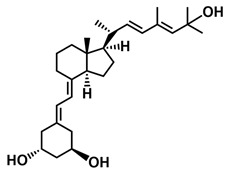	HL-60, MV4–11—human promyelocytic leukemia cells (antiproliferative and differentiation activity [59,100] HT-29—human colon cancer cells (antiproliferative activity) [59] MCF-7, T47D—human breast cancer cells (antiproliferative activity) [59]
PRI-5202	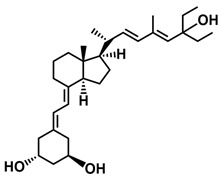	HL-60, MV4–11—human promyelocytic leukemia cells (antiproliferative and differentiation activity) [59,100] HT-29—human colon cancer cells (antiproliferative activity) [59] MCF-7, T47D—human breast cancer cells (antiproliferative activity) [59]
PRI-5105	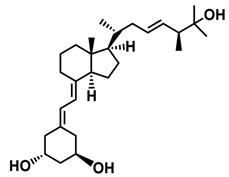	HL-60, MV4–11—human promyelocytic leukemia cells (antiproliferative and differentiation activity) [59] HT-29—human colon cancer cells (antiproliferative activity) [101]
PRI-5106	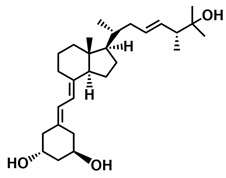	HL-60, MV4–11—human promyelocytic leukemia cells (antiproliferative and differentiation activity) [59] HT-29—human colon cancer cells (antiproliferative activity) [101]

### 7.3. Carborane Analogues of 1α,25(OH)_2_D_3_

Carboranes are highly lipophilic carbon–boron clusters that make proton–proton bonds. For this reason, carboranes were used as pharmacophores in biologically active molecules and boron neutron capture therapy (BNCT) to selectively kill cancer cells. Carborane analogs of 1,25(OH)_2_D_3_ (Table 3) were reported to be promising compounds with interesting biological activities [106,107,108]. In these analogs, the carborane substituent is located at the end of the side chain or it replaces the D-ring of 1,25(OH)_2_D_3_. 

### 7.4. CD-Ring-Modified Analogs with Anticancer Activity

Even a large modification in the CD-ring structure of a vitamin D analog does not decrease its activity if the 3D arrangement of the three hydroxyls at C-1, C-3, and C-25, responsible for binding to the VDR is retained [33,34]. Modifications to the CD ring led to a significant reduction or the total removal of calcemic action. At the same time, these modifications contributed to the development of analogs that selectively induced the differentiation of cancer cells.

The first analogs lacking the CD ring, named retiferols, were reported as early as 1995. *des*-CD analogs contain in their structure a cyclohexane A-ring with 1α-hydroxyl and an aliphatic chain. Analogs structured in this way mimic both cholecalciferol (vitamin D) and the retinoid backbone. They were developed based on a hypothesis that the CD-ring originating from the steroid backbone might not be necessary for activity. Since the preparation of the first retiferol, RAD2, several *des*-CD analogs have been synthesized and their activity has been investigated [33,34]. The combination of specific properties of vitamin D and retinoids in the same hybrid molecule that is practically non-toxic might represent an important avenue to new cancer therapeutics. The 13,13-dimethyl-*des*-CD analog **2** (Table 4) of (20*S*)-1,25-dihydroxy-2-methylene-19-*nor* vitamin D_3_ showed vitamin D-like biological activity and retained a high affinity for the VDR [109].

A group of new analogs lacking the C-ring, with an *m*-phenyl aromatic ring to mimic the D-ring and a 6-carbon hydroxylated side chain attached to the aromatic nucleus or triene system or the newest compounds with either a rigidified alkyne side chain or fluorine atoms at the terminus of the side chain, showed anti-proliferative and differentiating activity on human breast adenocarcinoma cells. Of great importance is the lack of in vivo calcemic activity in this group of analogs [14,35,110].

## 8. Conclusions

1,25(OH)_2_D_3_ is a seco-steroid hormone with a broad spectrum of activity, affecting the function of many systems in the human body. Of particular interest is the antiproliferative/differentiating action of 1,25(OH)_2_D_3_ and its analogs against cancer cells. The only clinically relevant adverse effect of therapeutic doses of 1,25(OH)_2_D_3_ is hypercalcemia, leading to tissue calcification. In vitro studies using human cell lines, including colorectal cancer (HT-29) and promyelocytic leukemia (HL-60, MV4–11), and laboratory animal experiments have demonstrated significant antiproliferative and cell differentiation-inducing effects of the synthetic analogs of 1,25(OH)_2_D_3_. The compounds used had a reduced effect regarding increasing blood calcium levels, as compared with 1,25(OH)_2_D_3_. Therefore, analogs of 1,25(OH)_2_D_3_ may play an important role in the development of novel anti-cancer therapies. The rational design of newer and more potent compounds is hindered because our current knowledge of the structural interactions between agonists and the VDR is based on information from studies using an artificial truncated VDR. The structure of the full-length native VDR is still lacking and in need of a resolution. Therefore, the next steps to moving forward with the biologically active molecules described in this review are to understand how they interact with the VDR LBD, make use of the native VDR, and solve the crystal structures of the analogs with which they are complexed. Of interest is the conformation that analogs adopt in the VDR LBP, how they affect the structure of the VDR helixes, particularly of helix H12, and how they interact with the amino acid residues of the VDR LBD. Molecular modeling and quantum mechanical calculations have an important role to play in revealing the complex relationship between interactions at the molecular level and the biological activity of analogs. The outcomes of such theoretical experiments for the existing agonists are necessary for the rational design of new VDR agonist structures with increased affinity for the VDR and enhanced anticancer activity.

## Figures and Tables

**Table 1:** Vitamin D metabolites and analogs of therapeutic importance.

Structure and Abbreviated Name	International Nonproprietary Name	Therapeutic Use
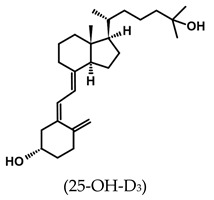	calcidiol	chronic hypocalcemia, renal osteodystrophy, rickets
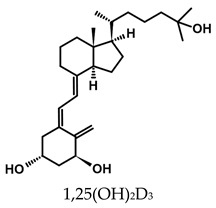	calcitriol	renal osteodystrophy, osteoporosis, psoriasis
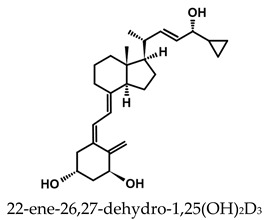	calcipotriol	psoriasis
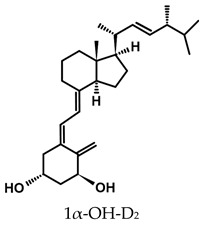	doxercalciferol	secondary hyperparathyroidism
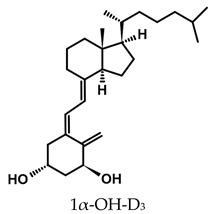	alfacalcidol	renal osteodystrophy, secondary hyperparathyroidism, osteoporosis, rickets
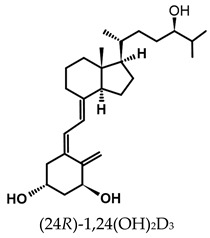	tacalcitol	psoriasis
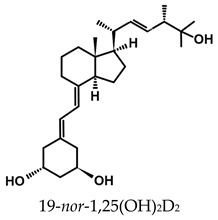	paricalcitol	secondary (renal) hyperparathyroidism
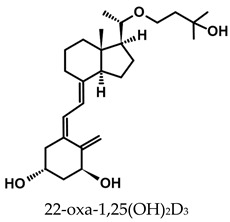	maxacalcitol	psoriasis, secondary hyperparathyroidism
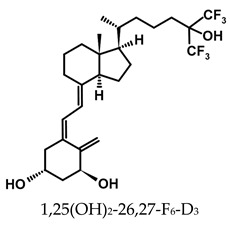	falecalcitriol	secondary hyperparathyroidism, osteoporosis
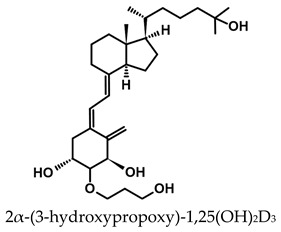	eldecalcitol	osteoporosis

**Table 3:** Carborane analogues of 1,25(OH)_2_D_3_.

Compound	Structure	Classification/Type of Cancer (Biological Activity)
**1**	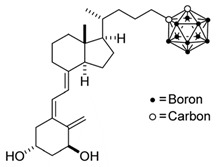	MCF-7—human breast cancer cells (antiproliferative activity) [108]
**2**	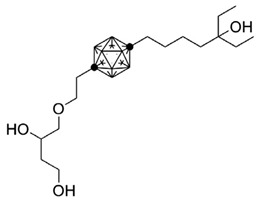	HL-60—human promyelocytic leukemia cells (differentiation activity) [106,107]
**3**	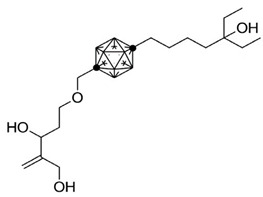	HL-60—human promyelocytic leukemia cells (differentiation activity) [106]
**4**	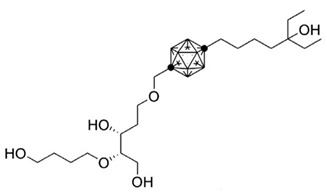	HL-60—human promyelocytic leukemia cells (differentiation activity) [106]

**Table 4:** CD-ring-modified analogs with differentiating activity.

Compound	Structure	Classification/Type of Cancer (Biological Activity)
**1**	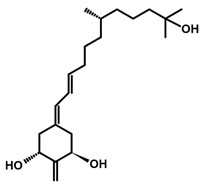	HL-60—human promyelocytic leukemia cells (differentiation activity) [109]
**2**	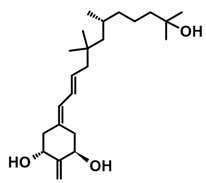	HL-60—human promyelocytic leukemia cells (differentiation activity) [109]
**3**	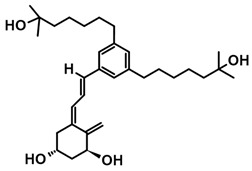	MCF-7—human breast cancer cells (antiproliferative and differentiation activity) [35]
**4**	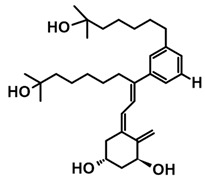	MCF-7—human breast cancer cells (antiproliferative and differentiation activity) [35]
**5**	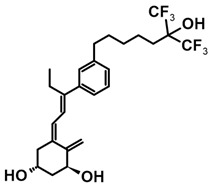	MDA-MB-231—human breast cancer cell line (antiproliferative activity) [110]
**6**	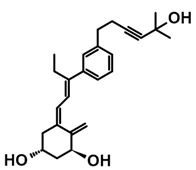	MDA-MB-231—human breast cancer cell line (antiproliferative activity) [110]
**7**	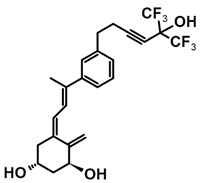	MDA-MB-231—human breast cancer cell line (antiproliferative activity) [110]
